# A Module Analysis Approach to Investigate Molecular Mechanism of TCM Formula: A Trial on Shu-feng-jie-du Formula

**DOI:** 10.1155/2013/731370

**Published:** 2013-11-26

**Authors:** Jianglong Song, Fangbo Zhang, Shihuan Tang, Xi Liu, Yibo Gao, Peng Lu, Yanping Wang, Hongjun Yang

**Affiliations:** ^1^Institute of Automation, Chinese Academy of Sciences, Beijing 100190, China; ^2^Institute of Chinese Materia Medica, China Academy of Chinese Medical Sciences, Beijing 100700, China; ^3^Institute of Basic Research in Clinical Medicine, China Academy of Chinese Medical Sciences, Dongzhimen, Beijing 100700, China

## Abstract

At the molecular level, it is acknowledged that a TCM formula is often a complex system, which challenges researchers to fully understand its underlying pharmacological action. However, module detection technique developed from complex network provides new insight into systematic investigation of the mode of action of a TCM formula from the molecule perspective. We here proposed a computational approach integrating the module detection technique into a 2-class heterogeneous network (2-HN) which models the complex pharmacological system of a TCM formula. This approach takes three steps: construction of a 2-HN, identification of primary pharmacological units, and pathway analysis. We employed this approach to study Shu-feng-jie-du (SHU) formula, which aimed at discovering its molecular mechanism in defending against influenza infection. Actually, four primary pharmacological units were identified from the 2-HN for SHU formula and further analysis revealed numbers of biological pathways modulated by the four pharmacological units. 24 out of 40 enriched pathways that were ranked in top 10 corresponding to each of the four pharmacological units were found to be involved in the process of influenza infection. Therefore, this approach is capable of uncovering the mode of action underlying a TCM formula via module analysis.

## 1. Introduction

With the development and evolution for thousands of years, Traditional Chinese Medicine (TCM) has become a sound and complete theory based on distinct principles and foundation from Western Medicine. TCM formulae, characterized by abundant ingredients and vast associated targets, are usually effective alternatives to western drugs for various multifactorial disorders [1]. Influenced by the decreased efficiency of new drug invention in recent years, the pattern of drug design has to evolve from traditional “one drug, one target” to “multicomponent, multitarget” drug discovery [2, 3]. As multicomponent agent with potential treatment effects, TCM formula holds great promise to promote the process of multitarget drug discovery based on molecular networks [1, 4]. Thus, the investigation of molecular mechanism of TCM formula plays an important role for better understanding the essence of TCM therapies and multicomponent drug discovery.

Currently, network-based approaches become crucial in unveiling and interpreting the mode of action of a TCM formula, with the accumulation of volume “omics” data and the emerging of network pharmacology. So far, lots of researchers have made great effort to acquire and collect “omics” data through advanced in vivo and in vitro techniques [5–7]. Among various “omics” data, interaction knowledge such as compound-protein interaction (CPI) and protein-protein interaction (PPI), as well as Gene Ontology (GO) and pathway annotation, make it possible to describe and analyze complex TCM formula in a holistic manner by using computational techniques. On the other hand, network pharmacology brought new insight into drug discovery once it was put forth [8]. The research interests of drug discovery extend from simple disease-drug-gene relations to some new spots such as promiscuity, synergistic effect, and functional modules [9–11]. Consequently, the focus in pharmacology research has shifted to the exploration of multicomponent multitarget drugs [3, 12]. In fact, plenty of work investigated the intrinsic regulating mechanism between many drugs and numerous targets or synergistic effects of drug combinations from a network perspective [13, 14]. Meanwhile, numerous network-based methods have been developed to decipher the pathological pattern underlying complex disorders and uncover the mode of action of TCM herbs or formulae [15, 16]. Moreover, network target was introduced as a new subject for studying the pharmacological action of TCM herbs rather than individual target or target set [17]. By using network-based techniques, several TCM formulae such as Liu-wei-di-huang have been particularly observed and studied in order to discover the underlying mode of action at the molecular level [18]. Therefore, it is essential to investigate the molecular mechanism of TCM formula using network-based methods, especially in TCM pharmacology research [1].

Notably, module analysis technique based on network model holds great promise to deal with most widely-used TCM formulae of unexpected complexity at the molecular level. In general, a TCM formula contains hundreds of chemical constituents and may associate with thousands of potential targets. It is a challenging task to identify the effective bioactive compounds or even discover the pharmacological action of numerous constituents of a TCM formula [1]. Thus, it is of great importance to capture the dominant modules of the molecular network representing a TCM formula. Two common types of dominant modules we are interested in are functional module and pharmacological unit. A functional module usually represents a group of genes or proteins sharing similar molecular functions, while a pharmacological unit is a connected subnetwork in which a set of compounds with similar physiochemical properties modulate the activities of a group of function-similar gene products. Typically, functional modules or pharmacological units within the molecular network usually hold some significant properties that are helpful in revealing the mode of action of TCM formula. In fact, numbers of researchers proposed diverse methods to detect functional modules from interaction networks [11, 19, 20]. On the contrary, there are few researches on identifying pharmacological units for multicomponent drugs [18]. On the other hand, network clustering algorithms, also known as module detection methods, developed from statistical physics are usually capable of finding significant communities enriched with explicit real-world meaning [21, 22]. As a matter of fact, several algorithms accomplished important tasks in biological field such as identifying protein complexes [23–25]. Additionally, to identify functional modules or pharmacological units is obviously another application of network clustering methods, which is crucial in the investigation of pharmacological action of TCM formula. Hence, applying classic module detection algorithms to the molecular network of TCM formula may contribute to better understanding of its mode of action at the molecular level.

We here proposed a computational approach combining clustering algorithm with heterogeneous network to investigate the molecular mechanism of TCM formula. This approach takes a three-step procedure. At first, we constructed a 2-class heterogeneous network (2-HN) comprised of herbal ingredients and associated targets for a TCM formula under study. Then, a classic module detection algorithm was applied to the 2-HN and we identified pharmacological units from the 2-HN. Finally, we finely selected primary pharmacological units and investigated them by pathway analysis. This approach is apparently applicable for any TCM formula. In this paper, we use Shu-feng-jie-du formula (SHU formula) as an example to illustrate the procedure of the approach. The pathway analysis of four pharmacological units identified from the 2-HN for SHU formula showed that 24 out of 40 enriched pathways that were ranked in top 10 corresponding to each of the four pharmacological units were directly or indirectly involved in the process of influenza development.

## 2. Methods

The novel approach is aimed at discovering the molecular mechanism of TCM formula based on a heterogeneous network together with a clustering algorithm. The procedure of this approach mainly consists of three steps: the construction of a heterogeneous network, module detection from the network, and the pathway analysis of selected primary pharmacological units. In practice, we investigated the mode of action of Shu-feng-jie-du formula by using this approach.

### 2.1. Construct Heterogeneous Network

Since our approach takes advantage of the network to study a TCM formula, we should firstly construct a heterogeneous network comprised of herbal ingredients and potential targets. At the beginning, the specific composition of each herb in a given TCM formula must be acquired. Typical ways to collect the chemical ingredients of herbs include literature mining, TCM database retrieval, and identification test. By diverse means, we can collect the chemical constituents together with their geometric structure for all herbs in the TCM formula. Subsequently, the interaction data and potential targets could be computed and retrieved, respectively, based on the chemical knowledge for the studied TCM formula.

First of all, we acquire the interaction data between herbal compounds by computational chemistry techniques. Although various kinds of interaction knowledge is available, compound pairs with similar chemical structures are widely used in network-based pharmacology and drug discovery research. The rationale is a well-known assumption that similar compounds have similar properties [26]. In other words, similar chemicals may share common targets and are likely to perform synergistic action on complex diseases. Thus, we evaluate compound pairs by calculating the pairwise chemical similarity using the geometric structure previously curated. In the field of cheminformatics, various methods were proposed to compute the structural similarity between compounds. Notably, fingerprint-based similarity is practically preferable to Maximum Common Subgraph (MCS) and other methods in dealing with a large number of compound pairs. We here employ Pybel, a Python wrapper for the Openbabel toolkit, to calculate fingerprint-based chemical similarity [27]. In the similarity measure, Tanimoto Coefficient is used to evaluate the commonness of fingerprints derived from two corresponding compounds as follows:

(1) $$\begin{array}{c} s_{\text{TC}}\left(c_{1},c_{2}\right)=\frac{c}{a+b-c}, \end{array}$$

where *c*_1_ and *c*_2_ are two compounds; *a* and *b* are bit lengths of *c*_1_ and *c*_2_ fingerprints, respectively; and *c* is the number of common bits between *c*_1_ and *c*_2_ fingerprints. In addition, a threshold *θ* is predefined to determine whether two compounds are similar in structure. Compound pairs are considered to be similar only if the pairwise similarity is equal to or greater than the threshold. In the end, similar compound pairs are collected as one of the sources for the construction of the heterogeneous network.

Next, we retrieve potential targets from some authentic databases according to the chemical constituents within the TCM formula under study. When retrieving a specific database such as DrugBank, CTD, and STITCH, we regard the gene products that interact with herbal compounds as potential targets. Note that only gene products of homosapiens (human) will be taken into consideration. Once the initial set of potential targets is achieved, the potential targets should be carefully selected in order to avoid contingency. It is understood that “hub” targets usually associate with two or more chemicals due to the promiscuous property of potential target in pharmacological space [9, 13]. So we here define Promiscuity Index of a target simply by the number of chemicals interacting with that target. Similarly, the Promiscuity Index of a chemical can be measured by the number of its binding targets. A threshold *δ* is specified beforehand to eliminate peripheral targets curated for the TCM formula. Gene products with Promiscuity Index no less than *δ* are eventually selected into the target set for the TCM formula. Note that the threshold *δ* is a small integer but is greater than one, for instance, 2 or 3.

Then, we collect interaction relations between gene products in the target set from some authentic databases. Recent findings demonstrated that proteins always function in cooperation with others rather than in isolation inside or out of a cell [13]. That is, gene products tend to form functional modules to participate in certain biological processes or accomplish specific physiological functions. Lots of databases, such as HPRD, BioGrid, IntAct, and DIP, gather plenty of acknowledged protein-protein interactions (PPIs) across diverse species. We usually select one database as the source of PPI data due to the diverse reliability of PPIs in different databases. Therefore, the interactome knowledge is introduced to the heterogeneous network by retrieving PPIs between gene products in the target set.

**Figure 1:**
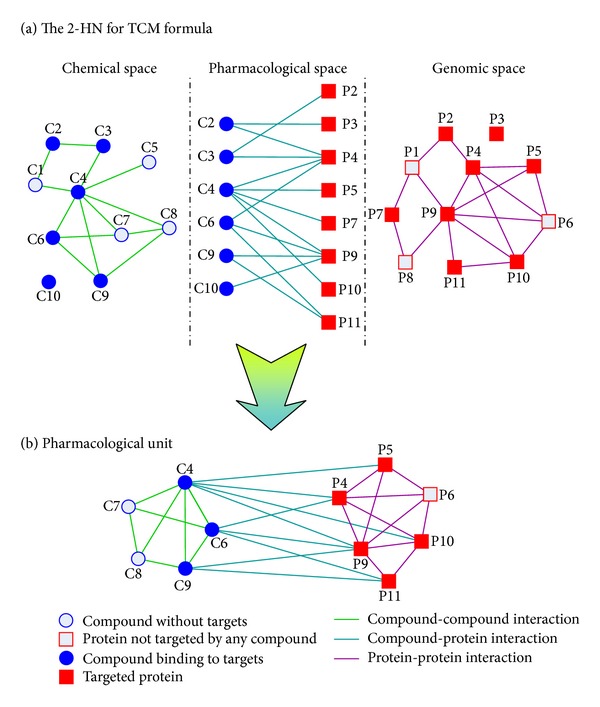
(a) A 2-class heterogeneous network (2-HN) modeling the complex system of a TCM formula and its molecular targets. A 2-HN can be simply divided into three subnetworks in chemical, pharmacological, and genomic space in terms of the type of links. (b) A pharmacological unit identified from the 2-HN in (a). A pharmacological unit includes a set of structure-similar herbal compounds and a group of function-similar target genes, indicating that the herbal compounds modulate the activities of gene products.

Finally, we construct an integrated network on the basis of heterogeneous data acquired before. Since compounds and gene products are present in this integrated network at the same time, we consider such a network as a 2-class heterogeneous network (2-HN). In brief, 2-class heterogeneous network (2-HN) is an abstract network model involving two distinct groups of objects. As a matter of fact, heterogeneous network, sometimes viewed as multilayer network, has been employed in recent work to study complex drug-target interactions and predict disease genes [18, 28, 29]. In our case, the 2-HN describes a complex pharmacological system relating the TCM formula under study to its treatable diseases. From a local point of view, the 2-HN can be divided into three subnetworks in chemical, pharmacological and genomic space in terms of three types of links in the 2-HN (Figure 1) [30]. In most cases, it is difficult to investigate and analyze the 2-HN for the TCM formula due to its complexity. Moreover, dense modules identified from the 2-HN may reveal some important pathways enriched in a subset rather than the whole set of genes related to the TCM formula. Therefore, to identify the pharmacological units from the 2-HN by module detection methods is always necessary to uncover the molecular mechanism of the TCM formula (Figure 1).

### 2.2. Detect Significant Modules

Since the complex network theory emerged, module detection has become one of the major techniques to promote the application and development of complex network. A great quantity of algorithms have been devised and implemented to find significant modules from connected networks [21–23]. Among various classic methods, a well-known method, Girvan-Newman algorithm, is capable to detect communities of a complex system and identify community structure [22]. Girvan-Newman algorithm is performed by iteratively removing edges with highest betweenness from the original network. In this way, the community structure could be viewed as a dendrogram. We employ clusterMaker, an implementation of Girvan-Newman algorithm in Cytoscape, to identify significant modules within the 2-HN for the TCM formula [31, 32].

After the clustering partition is detected from the network, we need a measure to quantify the significance of identified modules. Notably, modularity is an outstanding quality function measuring the goodness of network partition [33, 34]. Consequently, we use a measure similar to the definition of modularity to evaluate whether a module is significant or not in the original network. For an undirected simple graph, the modularity of a module *C* can be expressed as follows:

(2) $$\begin{array}{c} Q\left(C\right)=\frac{l_{C}}{m}-{\left(\frac{d_{C}}{2m}\right)}^{2}, \end{array}$$

where *l*_*C*_ = ∑_*ij*∈*C*_ *w*_*ij*_ is the summation of weights of edges in module *C*; *d*_*C*_ = ∑_*n*∈*C*_deg⁡(*n*) is the summation of degrees of nodes in module *C*; and *m* = (1/2)∑_*i*,*j*∈*G*_ *w*_*ij*_ is the size of the graph *G*. Obviously, a significant module corresponds to a modularity larger than zero. A “good” module always has a large modularity; otherwise, a small modularity indicates the “poor” significance of a network module. Moreover, according to the definition above, the modularity of a clustering partition of a given network is just the summation of modularities over all modules in the partition.

### 2.3. Analyze Pharmacological Units

The significant modules identified from the 2-HN of the TCM formula need to be examined before conducting further analysis. First, modules should be excluded if they are only comprised of compounds or gene products. Since compound-protein interactions (CPIs) relate herbal ingredients to potential targets, modules without any CPI make little contribution to uncover the pharmacological action of herbal compounds in the TCM formula. Second, modules with small modularity close to zero should be eliminated. Generally, a module may not be significant enough to be considered as a rational pharmacological unit for the TCM formula if it has a fairly small modularity. Third, modules should be paid less attention if the ratio of preserved compound-protein interactions is particularly low. The ratio of preserved CPIs is defined as the number of CPIs in a module divided by the total number of CPIs in the 2-HN. The ratio for a module *C* can be expressed as

(3) $$\begin{array}{c} R\left(C\right)=\frac{\left|\left\{e_{cg}\;|\;c,g\in C\right\}\right|}{\left|\left\{e_{cg}\;|\;c,g\in G\right\}\right|}, \end{array}$$

where *c* is a compound and *g* is a gene product; |·| is the norm of a set, that is, the number of elements in the set. If the ratio is low or few CPIs are present in a module, the module is unlikely to represent the primary interacting pattern that links herbal compounds and potential targets for the TCM formula under study. After these examinations, the remaining modules can be simply regarded as primary pharmacological units responsible for the studied TCM formula taking effect on complex diseases.

We investigate and analyze the primary pharmacological units by pathway analysis. Pathway analysis always play an essential role of discovering possible biological processes that the genes in the input list participate in. A lot of databases collect many curated pathways concerning metabolism, cellular processes, and diseases, such as KEGG, BioCarta, Reactome, GeneGo, and Ingenuity. Besides, Gene Ontology (GO), another kind of pathways, usually reveals the physiological functions and cellular locations of a group of genes or gene products. Thus, pathway and GO supply us with sufficient knowledge about molecular regulation and gene function. Other analysis methods, for instance, disease analysis using gene overlapping and biomarkers, could provide new insight to understand the underlying functions of the TCM formula. In this paper, we use MetaDrug, a platform of systems pharmacology and toxicity, to perform pathway analysis for the identified primary pharmacological units [35]. Then, the molecular mechanism underlying the studied TCM formula could be uncovered through analyzing the enriched pathways or GO terms for primary pharmacological units.

To illustrate the workflow of the approach in detail, we apply the approach to an effective agent for influenza, Shu-feng-jie-du formula. Instead of Shufeng-jie-du formula, we use SHU formula for short in following sections. Following the procedure of the approach, we can investigate the mode of action underlying SHU formula.

## 3. Results and Discussion

### 3.1. 2-HN for SHU Formula

We firstly acquired the herb composition of SHU formula and collected chemical constituents within each herb. In fact, SHU formula mainly consists of 8 herbs: Bai-Jiang-Cao (Herba Patriniae), Ban-Lan-Gen (Radix Isatidis), Chai-Hu (Radix Bupleuri), Gan-Cao (Radix Glycyrrhizae), Hu-Zhang (Rhizoma Polygoni Cuspidati), Lian-Qiao (Fructus Forsythiae), Lu-Gen (Rhizoma Phragmitis), and Ma-Bian-Cao (Herba Verbenae) (Table 1). According to the herb composition, we collected 243 nonredundant chemical constituents for this formula. All constituents of SHU formula were retrieved from the Chemistry Database founded by Shanghai Institute of Organic Chemistry (http://www.organchem.csdb.cn). The 2D structures of herbal constituents were downloaded from PubChem Compound database according to unique CAS Registry Number. Then, we evaluated the similar compound pairs based on the fingerprint-based Tanimoto similarity. The threshold *θ* for similarity score was set to 0.7 as stated in [27]. In this way, 562 pairs of compounds were collected and considered to be similar because they had comparable structural similarities to the threshold. In the next step, we searched Comparative Toxicogenomics Database (CTD) for potential targets interacting with herbal ingredients in SHU formula [36]. The threshold *δ* for Promiscuity Index of potential targets was set to 3. Namely, we only selected gene products targeted by at least 3 herbal compounds, as well as the interactions between those proteins and chemicals. As a result, 238 potential targets were collected from CTD, which associated with herbal compounds by 1101 interactions. At last, we extracted acknowledged interactions between 238 gene products extracted before from BioGRID database [37]. There were 718 nonredundant PPIs between the curated potential targets. Based on these data, a 2-HN, an integrated network for SHU formula, was constructed. Since we focused on the largest connected component of the 2-HN for SHU formula, the resultant network contained 171 herbal compounds and 238 potential targets after discarding small-size components (Table 2).

The 2-HN of SHU formula has some interesting properties in topology. As shown in Table 2, two groups of nodes in the 2-HN (rectangle for compounds and ellipse for gene products) are connected by three types of links. It is obvious that the pharmacological subnetwork is a bipartite, which is comprised of all CPIs (Table 2). So the 2-HN for SHU formula is beyond a bipartite by including compound interactions and PPIs (Table 2). The network heterogeneity decreases from 2.531 of the pharmacological subnetwork to 1.588 of the 2-HN for SHU formula. This is because compound interactions and PPIs bring many extra links to the “nonhub” chemicals and gene products, respectively [38]. In addition, the chemical subnetwork has 34 connected components of which 17 are isolated compounds (Table 2). Regardless of the isolated nodes, each of the remaining connected components has 9.059 compounds in average. That is, herbal compounds in SHU formula tend to form multiple components in terms of similar structure. As for the genomic subnetwork, there are 57 connected components, among which 55 are comprised of isolated proteins (Table 2). In fact, nearly all of the nonisolated proteins connect to a giant component with 181 nodes and 717 links in the genomic subnetwork. It suggests that the giant component determines the mode of action of SHU formula to a large extent. Different from the phenomenon in chemical subnetwork, target proteins of SHU formula tend to form a single large component instead of multiple components. Furthermore, only a small fraction (50 out of 171) of the involved herbal compounds (blue rectangles) take direct or indirect actions on the 238 gene products in the 2-HN (Table 2). Apart from the incompleteness of chemical-protein knowledge, we could see that only limited number of compounds have acknowledged therapeutic effects in SHU formula. Among these 50 compounds, there are several “hub” compounds associated with many targets, such as quercetin and resveratrol, which may exhibit high activities against influenza progression.

The “hub” compounds usually play an essential role to achieve the excepted effect of SHU formula treating influenza. We listed four “hub” herbal compounds in Table 3 and investigated their pharmacological functions at the same time. Two outstanding compounds are quercetin and resveratrol with far larger Promiscuity Index (222 and 218, resp.) than other compounds (the third largest is 67 for kaempferol). Previous works revealed the underlying functions of these four compounds in defending against influenza. For instance, quercetin could relieve the oxidative stress caused by experimental influenza virus infection in organisms like lungs and liver [39]. Another work demonstrated that quercetin together with oseltamivir exhibited antivirus effect on the Toll-like receptor 7 (TLR7) signaling pathway when dendritic cells and macrophages were infected with H1N1 [40]. Several quercetin derivatives such as quercetin-3-rhamnoside and isoquercetin also served as anti-influenza agents by inhibiting the replication of influenza virus [41, 42]. Additionally, resveratrol was found to inhibit the replication of influenza virus in MDCK cells, which involved the blockade of the nuclear-cytoplasmic translocation of viral ribonucleoproteins [43]. Moreover, kaempferol could inhibit the influenza A nucleoprotein production in human lung epithelial cells infected by the H5N1 virus [44] and eugenol could inhibit autophagy and influenza A virus replication by suppressing the activation of ERK, p38MAPK, and IKK/NF-*κ*B signal pathways [45]. Therefore, these four “hub” herbal compounds, characterized by large Promiscuity Index, indeed take effect to defend against influenza.

Although the general effect of SHU formula could be observed by studying the “hub” herbal compounds in the 2-HN, we still needed module analysis to further investigate the biological pathways that SHU formula actually influences and regulates. We firstly identified primary pharmacological units from the 2-HN for SHU formula and then investigated the particular mode of action of SHU formula treating influenza.

### 3.2. Pharmacological Units from the 2-HN

Through detecting modules using Girvan-Newman algorithm, 12 significant modules were identified from the 2-HN for SHU formula. However, not all the modules are fairly important and need to be analyzed in detail. We selected primary pharmacological units from the 12 modules according to three principles explained before. As shown in Table 4, module 11 is only comprised of compounds and thus excluded because it is not a valid pharmacological unit (including compounds and gene products). We chose 0.02 as the threshold for modularity and consequently five more modules, 7, 8, 9, 10, and 12, were discarded due to the low significance in the original network. The threshold for the ratio of preserved CPIs was set to 0.01 and another two modules, 3 and 6, were eliminated as they included too few CPIs. In the end, four modules, 1, 2, 4, and 5, were selected and considered as primary pharmacological units. From the topological perspective, modules 1, 2, 4, and 5 are highly connected in the background network of the 2-HN characterized by relatively large modularities. Besides, these four pharmacological units are of great importance to represent the pharmacological essence of SHU formula due to the large amount of preserved CPIs from the original system. So we made great effort to investigate these four pharmacological units by pathway analysis.

**Figure 2:**
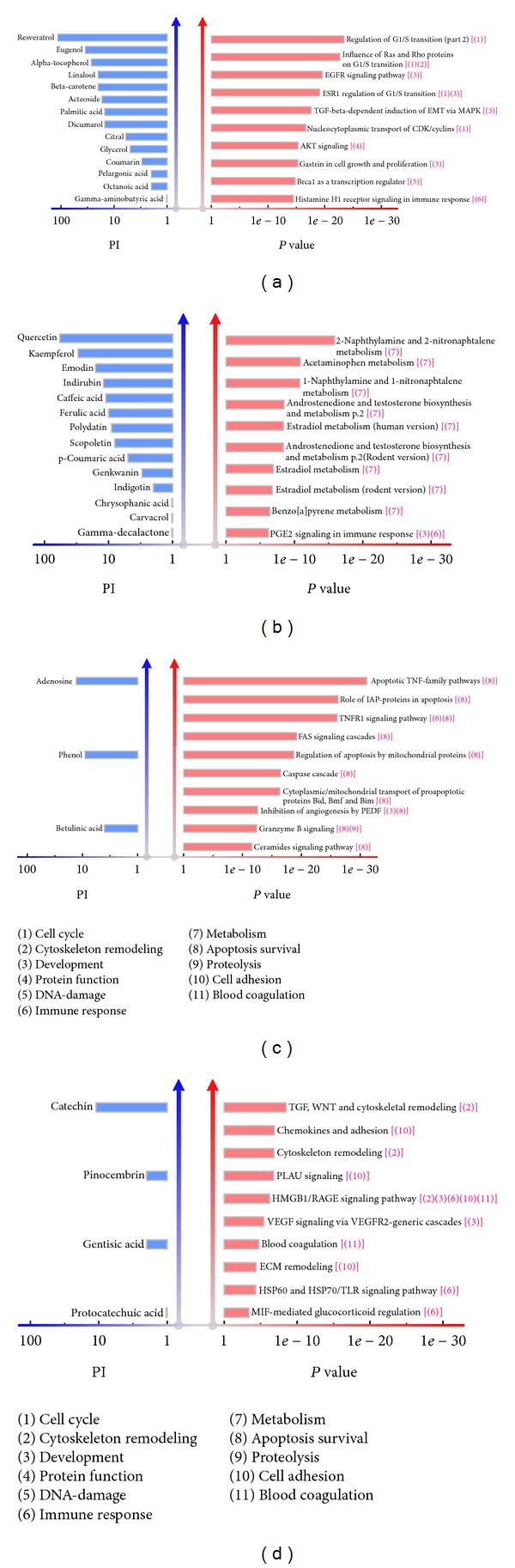
(a), (b), (c), and (d) Top 10 enriched pathways and associated herbal compounds corresponding to module 1, 2, 4, and 5, respectively. The herbal compounds are ranked by Promiscuity Index (PI), which is defined as the number of targets connected to a given compound by the preserved CPIs in a detected module. Note that only compounds with PI greater than zero are listed in this figure. The enriched pathways are ranked by the *P* values calculated in MetaDrug. The circled numbers in brackets after pathway name indicate the major category that pathway belongs to. For example, “ESR1 regulation of G1/S transition” belongs to category 1 and 3, that is, cell cycle and development. The category knowledge is curated from the classification tree of GeneGo pathways in MetaDrug. All pathways in this figure are significant with *P* values lower than 0.001.

We analyzed the underlying biology by performing enrichment analysis with pathways from GeneGo database. For each primary pharmacological unit, we employed the genes within the module as input gene list to search for enriched pathways in GeneGo database. The top 10 enriched pathways corresponding to each module were illustrated in Figure 2. The pathways were sorted according to the *P* value which measured the significance of a given pathway enriched in the gene list of a pharmacological unit. The bioactive compounds in every pharmacological unit potentially acting on the enriched pathways were also highlighted in Figure 2. The associated herbal compounds were ranked by Promiscuity Index, which was defined as the number of targets connected to a given compound by the preserved CPIs in an identified module (Materials and Methods). From the viewpoint of pathway category, the bioactive compounds in every primary pharmacological unit seemed to particularly interfere with pathways from one or two specific categories. For example, compounds in module 1 generally participate in the processes of cell cycle (4 pathways) and development (4 pathways); the highly enriched pathways of module 2 exhibit high relevance to metabolism (9 pathways), especially the estradiol metabolism (3 pathways); module 4 mostly influence the biological processes related to apoptosis and survival (10 pathways); and module 5 interfere in the activities of cell adhesion (4 pathways) and cytoskeleton remodeling (3 pathways) as well as immune response (3 pathways). Despite of the redundancy of GeneGo pathways, we could see that each of the four pharmacological units tends to regulate relevant pathways from specific categories, which implies that SHU formula carries out pharmacological efficacy by simultaneously intervening pathological activities from distinct aspects at the pathway level. Since the module analysis approach was applied to SHU formula generated explicit results as exhibited in Figure 2, we should verify the reliability of the prediction and evaluate the relevance of SHU formula to influenza infection.

According to Figure 2, we could find that compounds in all four pharmacological units had potential effects on influenza infection. At first, 40 enriched pathways in Figure 2 were regulated to some extent by corresponding herbal compounds in each module, which can be explained by the acknowledged regulatory relations between compounds and pathway components from CTD. For example, resveratrol influences the EGFR signaling pathway through binding to EGFR protein and thus decreasing the phosphorylation of EGFR protein [46]. However, since not all enriched pathways were involved in the activities of influenza infection, we particularly focused on those related to influenza progression and the regulatory relations between SHU formula and those pathways. As shown in Table 5, 24 of the 40 enriched pathways were found to directly or indirectly participate in the processes of influenza virus invasion, production, proliferation, and transition, and to account for the influenza-induced syndromes as well, such as inflammation. Here we primarily studied the specific action of herbal compounds in each pharmacological unit on 24 influenza-related pathways, while the participation of these pathways in the progression of influenza would be analyzed in following section. For module 1, resveratrol together with other compounds blocked the G1/S-phase transition [47], inhibited the EGFR/HER2 signaling pathway [46], and regulated the PTEN/AKT pathway [46]. Quercetin and kaempferol together with other bioactive compounds in module 2 showed inhibitory effect on the in vitro hepatic metabolism of 17*β*-estradiol [48] and on the hydroxylation of benzo[a]pyrene [49]. Additionally, quercetin also suppressed COX-2 expression and PGE2 production [50]. Herbal compounds in module 4 such as adenosine, phenol, and betulinic acid tended to inhibit IL-12 and TNF-*α* production [51], downregulate the expression of IAP2 [52], and trigger CD95 (APO-1/Fas)- and p53-independent apoptosis [53]. Compounds in module 5 like catechin could inhibit the endotoxin-induced HMGB1 release [54] and block the TLR signaling pathway [55]. Moreover, the remaining 16 pathways were also likely to correlate with influenza infection, although there has been no literature support for those pathways so far. In brief, 24 influenza-related pathways elucidated the potential effects of SHU formula against influenza infection from diverse aspects at the pathway level.

Moreover, by exploring the development of influenza, we could explicitly see how the enriched pathways modulated by bioactive components in SHU formula led human physiological system to a serious disease state. These pathways either promoted the production and replication of viral RNAs or proteins or induced host immune response and inflammation. The participation of these pathways in the pathological process of influenza infection, discussed in the next section, explained how SHU formula treated against influenza infection by intervening various pathways in different stages and cellular locations.

### 3.3. SHU Formula Treating Influenza

When Influenza A virus (H1N1) enters host cells, it induces host cell cycle arrest in G(0)/G(1) phase and creates favorable conditions for viral replication. The nonstructural protein 1 (NS1) of influenza A virus induces G(0)/G(1) cell cycle arrest mainly through interfering with the RhoA/pRb signaling pathway, thus providing beneficial conditions for viral protein replication and accumulation [56]. The concentration and activity of RhoA protein is pivotal for G(1)/S phase transition, which were decreased with overexpressing NS1 [56]. When viral macromolecules interact with host proteins. High-mobility-group box (HMGB) proteins bind to the nucleoprotein (NP) component of viral ribonucleoproteins (vRNPs) in the absence of viral RNA, and HMGB1 protein plays a significant role in intranuclear replication of influenza viruses [74]. PI3K/Akt signaling pathway is activated by NS1 protein and inhibition of the PI3K/Akt pathway is an anti-influenza strategy which is still in an early phase of preclinical development [59]. In addition, influenza virus infection activates three distinct MAPKs, ERK, p38 MAPK, and JNK, to participate to various extents in the induction of PGE2 synthesis from arachidonic acid in human bronchial epithelial cells [64]. Metabolized benzo[a]pyrene (BaP) reduced viral IFN induction by approximately 80% assessed in LLC-MK2 cell [63].

Airway epithelium play an important role in host immune response. Many diverse viruses target a polarized epithelial monolayer during host invasion. The polarized epithelium restrict the movement of pathogens across the mucosa. This regulation can be attributed to the presence of a junctional complex between adjacent cells and to an intricate network of actin filaments [73]. Virus-infected alveolar epithelium regulate CCL2/CCR2-dependent monocyte transepithelial migration dependent on both classical beta(1) and beta(2) integrins but also junctional adhesion molecule pathways during influenza infection [72]. The epithelial response to inhaled pathogens in airway epithelium that deposit on the airway epithelial surface includes EGFR signaling cascades [57].

Influenza virus invasion is associated with host immunity and inflammation. Inflammatory cytokines such as TNF-*α*, IFN-*γ*, and ET-1 may trigger the occurrence of AMI [65]. Toll-like receptors (TLRs) play an important role in early, innate viral inhibition in naturally occurring influenza with inflammatory cytokine responses [75]. Histamine mediates the acute inflammatory and immediate hypersensitivity responses, and it has also been demonstrated to affect chronic inflammation and regulate several essential events in the immune response [61]. Type V collagen [col(V)] overexpression and IL-17-mediated anti-col(V) immunity are key contributors to obliterative bronchiolitis pathogenesis. IL-17 is shown to induce EMT, TGF-*β* mRNA expression, and SMAD3 activation, whereas downregulating SMAD7 expression in vitro [58]. Macrophage migration inhibitory factor (MIF) is involved in inflammatory responses to H5N1 influenza virus infections by induction of pulmonary inflammatory cytokines and chemokines [76]. BRCA1 regulates inflammation-induced endothelial cell function and limits endothelial cell apoptosis and dysfunction [60]. Pigment epithelial-derived factor (PEDF) suppresses inflammation by inhibiting lipopolysaccharide-driven macrophage activation in vitro and in vivo [68]. GzmB deficiency associated with pathology, morbidity, and mortality results in exacerbation of lymphocytic inflammation during bleomycin-induced acute lung injury [69]. Ceramide is the core of sphingolipid metabolism, and phosphorylation of ceramide by ceramide kinase gives rise to ceramide-1-phosphate which has also been shown to participate in inflammation [70].

Besides immune responses in host defence, influenza A virus infection induces endoplasmic reticulum stress, Fas-dependent apoptosis, and TGF-*β* production in a variety of cells [71]. Inhibitor of apoptosis proteins (IAPs) influence ubiquitin-dependent pathways that modulate innate immune signaling via activation of nuclear factor *κ*B (NF-*κ*B) [66]. Multiple influenza virus factors have been identified that can activate intrinsic or extrinsic apoptotic induction pathways. dsRNA, NS1, NA, and PB1-F2 are influenza virus inducers of apoptosis. dsRNA and NA act via an extrinsic mechanism involving proapoptotic host-defense molecules: PKR by induction of Fas-Fas ligand and NA by activation of TGF-beta. PB1-F2 act intrinsically by localization and interaction with the mitochondrial-dependent apoptotic pathway [67].

The symptoms of influenza virus infection are related to gender. Females suffer a worse outcome from influenza A virus infection than males, which can be reversed by administration of estradiol to females and reflects differences in the induction of proinflammatory responses [62].

### 3.4. Discussion

**Figure 3:**
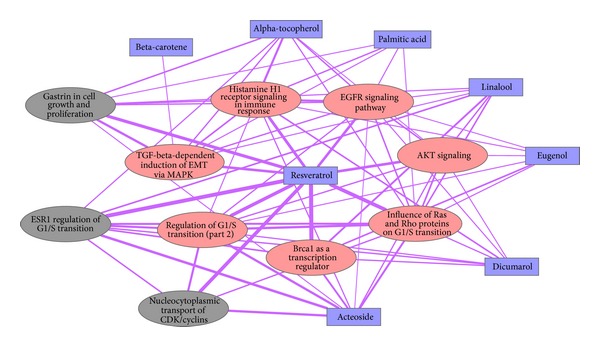
An illustration of SHU formula intervening the influenza development through multiple pathways. The blue rectangle is bioactive herbal compounds derived from SHU formula. The ellipse represents biological pathways that the compounds modulate. The red ones are literature-verified pathways that participate in the process of influenza infection, while the gray ones are not verified yet. A thick edge indicates many common hits (pathway components that are also associated targets of herbal compounds) between two pathways or between a compound and a pathway.

According to the results of pathway analysis, we built a simple network to illustrate the pharmacological action of SHU formula against influenza infection (Figure 3). This network was constructed based on module 1 identified by Girvan-Newman algorithm from the 2-HN of SHU formula. The edge connecting a compound and a pathway indicates the cooccurrence of associated targets of the compound and pathway components, while the edge between two pathways represents the commonness of hits (pathway components that are also associated targets of herbal compounds) corresponding to both pathways. As shown in Figure 3, 8 bioactive compounds of module 1 modulate 10 enriched pathways related to influenza infection. From the perspective of topology, resveratrol is the most important to regulate the involved pathways compared to other compounds. It is obvious that resveratrol is connected to all 10 pathways through strong links, indicating that resveratrol mediates multiple gene products in these pathways. Besides, resveratrol is found to modulate the *G*1/*S*-phase transition (*P* value 4.1*e* − 24) [47], the EGFR/HER2 signaling pathway (*P* value 2.8*e* − 20) [46], and the PTEN/AKT pathway (*P* value 5.3*e* − 16) [46]. Other compounds like Acteoside also perform similar functions on the involved pathways [77]. Of the top 10 enriched pathways, 7 (red ellipse) are found to participate in the development of influenza and its induced symptoms, illustrated in Table 5. Thus, the herbal compounds in Figure 3 are likely to intervene in the invasion, production, proliferation, and transition of influenza virus, through mediating multiple relevant pathways. Three pathways (grey ellipse) regulated by the compounds in Figure 3 hold great promise to influence the influenza development, while such prediction needs further work to test and verify.

In this paper, we presented a computational approach based on module analysis to investigate the molecular mechanism of TCM formula. This approach has several advantages. On one hand, we employed a precise model, 2-class heterogeneous network (2-HN), to represent the pharmacological system of a TCM formula. Since a 2-HN is structurally more complete than a bipartite by incorporating interactions within the same categories, so additional information is integrated into such a comprehensive model. In case of the 2-HN for SHU formula, besides the regulatory relations between chemicals and gene products, similar compounds within SHU formula and interactions between gene products are also taken into consideration when studying the mode of action of SHU formula. This additional information represented by compound-compound interactions (CCIs) and PPIs is critical to systematic investigation of multicomponent drugs, while traditional methods always disregard knowledge like this or use it separately [15]. On the other hand, the approach presented in this paper takes advantage of module detection technique to uncover the molecular mechanism of a TCM formula. Different from conventional methods, we analyze small-size yet topologically significant pharmacological units rather than the whole drug-target system of unexpected complexity. Generally, the pharmacological units identified by module detection methods are more reliable in topology than the original system. This is because the pharmacological units are significantly components in the original network featured by dense intraconnections. So a 2-HN together with module detection technique could deal with the challenging task of discovering the molecular mechanism of a TCM formula from its pharmacological system with hundreds of herbal compounds and thousands of targets, as well as unpredictable amount of interactions.

Although the approach provides new insight into molecular mechanism of TCM formula, it can be improved in three aspects. First, the compound interaction is not limited to structurally similar compound pair. The derivative or isometric relation, similarity in physicochemical property, and ontology similarity between compounds may outperform structural similarity to some extent. Second, the module detection methods could be improved in order to (i) identify modules with overlapping nodes and edges and (ii) take into account the differences of interactions in a 2-HN. Generally, a compound may have diverse therapeutic functions and a gene may participate in diverse biological processes. In other words, a node should be assigned to two or more modules representing diverse functions or processes. So overlapping modules detected from a 2-HN may be more consistent with reality. In addition, CPIs in a 2-HN should be paid more attention than CCIs and PPIs when detecting pharmacological units. This is because CPIs are indispensable in a pharmacological unit that is a connected subnetwork containing compounds and gene products. Third, we could adopt improved pathway analysis to uncover the biology underlying identified pharmacological units. As elaborated in [78], pathway enrichment analysis has two inevitable shortcomings. It treats every gene equally when finding pathways enriched in the input gene list. Besides, it does not take the pathway dependence into account, which results in three “Estradiol metabolism” pathways enriched in module 2 gene list (Figure 2). So precise pathway techniques are in need to find rational and reliable pathways underlying each primary pharmacological units from the 2-HN for a given TCM formula. With these improvements, the module analysis-based approach will be more capable of uncovering explicit molecular mechanism of TCM formula.

## 4. Conclusion

We here propose a computational approach based on module analysis to investigate the molecular mechanism underlying TCM formula. The approach incorporates the module detection technique with a 2-class heterogeneous network, a precise model to depict the complex system of a TCM formula. This approach mainly consists of three steps: network construction, module detection, and pathway analysis. The application of this approach to Shu-feng-jie-du formula outputs good results, which identified four primary pharmacological units uncovering key herbal compounds and essential pathways they modulated. 24 out of 40 enriched pathways that were ranked in top 10 corresponding to each of the four pharmacological units were found to be relevant to the process of influenza infection and some induced symptoms like inflammation. This demonstrates the effectiveness of our approach in discovering the molecular mechanism of a TCM formula. Although effective, this approach still requires improvement with regard to chemical similarity, module detection algorithm and accurate pathway analysis of identified modules. After all, our approach provides new insight into discovering the molecular basis of TCM formula and further promotes the large-scale exploration of the pharmacological action of multicomponent drugs in a low-cost manner, especially TCM formulae.

## Figures and Tables

**Table 1:** Herb composition of Shu-feng-jie-du formula (SHU formula).

English translation	Pharmaceutical name	Simplified Chinese script
Hu-Zhang	Rhizoma Polygoni Cuspidati	*虎杖*
Lian-Qiao	Fructus Forsythiae	*连翘*
Ban-Lan-Gen	Radix Isatidis	*板蓝* *根*
Chai-Hu	Radix Bupleuri	*柴胡*
Bai-Jiang-Cao	Herba Patriniae	*败酱* *草*
Ma-Bian-Cao	Herba Verbenae	*马鞭* *草*
Lu-Gen	Rhizoma Phragmitis	*芦根*
Gan-Cao	Radix Glycyrrhizae	*甘草*

**Table 2:** Topological properties of the 2-HN for SHU formula and its three subnetworks.

Property		CSN	PSN	GSN	2-HN
Node	Compounds	171	50	0	171
	Proteins	0	238	238	238
Edge	CCIs	481	0	0	481
	CPIs	0	1101	0	1101
	PPIs	0	0	718	718
Connected components		34	1	57	1
Isolated nodes		17	0	55	0
Clustering coefficient		0.662	0.0	0.198	0.414
Network density		0.033	0.027	0.025	0.028
Network heterogeneity		0.664	2.531	1.287	1.588

*CCI is short for compound-compound interaction, CPI is compound-protein interaction, and PPI is protein-protein interaction. CSN represents the chemical subnetwork of the 2-HN for SHU formula, PSN the pharmacological subnetwork, and GSN the genomic subnetwork.

*All the topological properties were calculated using Cytoscape 2.8 [32].

**Table 3:** “Hub” herbal compounds identified from the pharmacological subnetwork of the 2-HN for SHU formula.

Name	CAS RN	PubChem CID	PI	Action	Reference
Quercetin	117-39-5	5280343	222	(i) Quercetin and rutin exhibit prooxidant effect in healthy and antioxidant activity in influenza—infected animals.	[39]
				(ii) Quercetin and oseltamivir exhibited antivirus effect on the Toll-like receptor 7 (TLR7) signaling pathway when dendritic cells and macrophages were infected with H1N1.	[40]
Resveratrol	501-36-0	445154	218	Resveratrol inhibited the replication of influenza virus in MDCK cells.	[43]
Kaempferol	520-18-3	5280863	67	Kaempferol inhibited influenza A nucleoprotein production in human lung epithelial (A549) cells infected with the H5N1 virus strain A/Thailand/Kan-1/04 in non-toxic concentrations.	[44]
Eugenol	97-53-0	3314	61	Eugenol could inhibit autophagy and influenza A virus replication, inhibit the activation of ERK, p38MAPK and IKK/NF-*κ*B signal pathways.	[45]

*PI is Promiscuity Index of individual compound, that is, the number of binding targets in the 2-HN for SHU formula.

**Table 4:** Metrics of detected modules from the 2-HN for SHU formula.

Module	Compounds	Proteins	Valid	Modularity	Ratio of preserved CPIs
1	20	121	Yes	0.121375	0.257039
2	37	58	Yes	0.075361	0.15168
3	31	2	Yes	0.040522	0.003633
4	3	30	Yes	0.037876	0.023615
5	17	14	Yes	0.021214	0.014532
6	19	1	Yes	0.030336	0.001817
7	12	4	Yes	0.014417	0.003633
8	9	5	Yes	0.013261	0.004541
9	11	1	Yes	0.009457	0.000908
10	7	1	Yes	0.006564	0.000908
11	3	0	No	0.001104	0.0
12	2	1	Yes	0.000873	0.000908

**Table 5:** Literature-verified pathways related to influenza infection corresponding to four pharmacological units.

Module	Enriched pathways	*P*-value	Rank	Reference
1	Regulation of G1/S transition (part 2)	4.137*e* − 24	1	[56]
	Influence of Ras and Rho proteins on G1/S Transition	2.156*e* − 23	2	
	EGFR signaling pathway	2.803*e* − 20	3	[57]
	TGF-beta-dependent induction of EMT via MAPK	2.603*e* − 18	5	[58]
	AKT signaling	5.258*e* − 16	7	[59]
	Brca1 as a transcription regulator	1.710*e* − 15	9	[60]
	Histamine H1 receptor signaling in immune response	3.503*e* − 15	10	[61]

2	Estradiol metabolism (human version)	4.213*e* − 9	5	[62]
	Estradiol metabolism	1.293*e* − 7	7	
	Estradiol metabolism (rodent version)	1.832*e* − 7	8	
	Benzo[a]pyrene metabolism	4.024*e* − 7	9	[63]
	PGE2 signaling in immune response	6.146*e* − 7	10	[64]

4	Apoptotic TNF-family pathways	8.253*e* − 32	1	[65]
	Role of IAP-proteins in apoptosis	6.132*e* − 27	2	[66]
	FAS signaling cascades	6.374*e* − 20	4	[67]
	Inhibition of angiogenesis by PEDF	2.792*e* − 13	8	[68]
	Granzyme B signaling	3.712*e* − 13	9	[69]
	Ceramides signaling pathway	2.652*e* − 12	10	[70]

5	TGF, WNT, and cytoskeletal remodeling	3.303*e* − 9	1	[71]
	Chemokines and adhesion	1.360*e* − 7	2	[72]
	Cytoskeleton remodeling	1.502*e* − 7	3	[73]
	HMGB1/RAGE signaling pathway	5.901*e* − 7	5	[74]
	HSP60 and HSP70/TLR signaling pathway	4.805*e* − 5	9	[75]
	MIF-mediated glucocorticoid regulation	3.981*e* − 4	10	[76]

*The rank is the order of ascending *P* values of enriched pathways corresponding to each primary pharmacological unit.
